# Computer‐aided design files as a learning tool in dental anatomy

**DOI:** 10.1002/jdd.13439

**Published:** 2024-01-15

**Authors:** Elisandra Reyes‐Perez, Kendall Latshaw, Luiz Meirelles, Gabriela A. Weiss

**Affiliations:** ^1^ Division of Restorative and Prosthetic Dentistry, College of Dentistry The Ohio State University Columbus Ohio USA

## 1 PROBLEM

The COVID‐19 pandemic posed challenges to the dental curriculum,^1^ especially in areas where the concepts are traditionally learned through a hands‐on environment such as a preclinical laboratory setting. During the 2020 fall semester, a daunting concern was how dental schools could ensure continuity of education while safeguarding the health of students, faculty, staff, and patients. With the implementation of social distancing measures, in‐person learning was reduced and remote learning was instituted.^2^ An issue became evident during the first‐year Dental Anatomy course with the need to create a method for students to learn tooth morphology in a virtual setting.^3^, ^4^ The dental anatomy course establishes the foundation for many other classes in the dental school curriculum. Due to the intricacy and detail of learning tooth morphology, hands‐on learning and handling of extracted and typodont teeth is considered imperative when learning the course material.

## 2 SOLUTION

**FIGURE 1:**
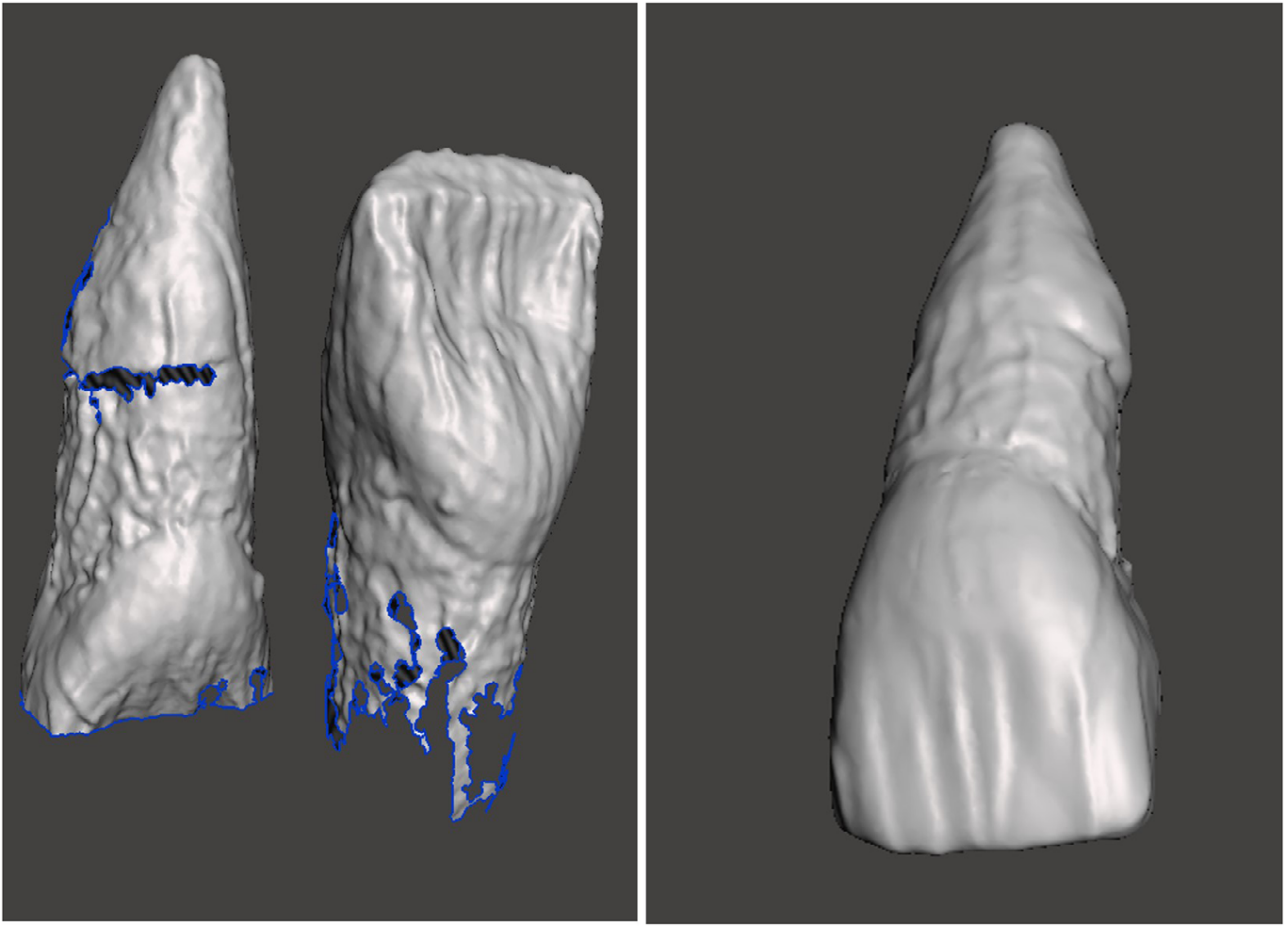
Scanning of the extracted teeth with the bench top scanner (Ceramill Map 600, Amann Girrbach). Coronal and apical halves of teeth were scanned and imported into Meshmixer. The files were then aligned, merged, reduced, and smoothed to create the final product that was exported as a .ply file.

A digital tooth library was created by scanning over 100 extracted teeth used in the dental anatomy course with a bench top scanner (Ceramill Map 600, Amann Girrbach, see Figure 1).

**FIGURE 2:**
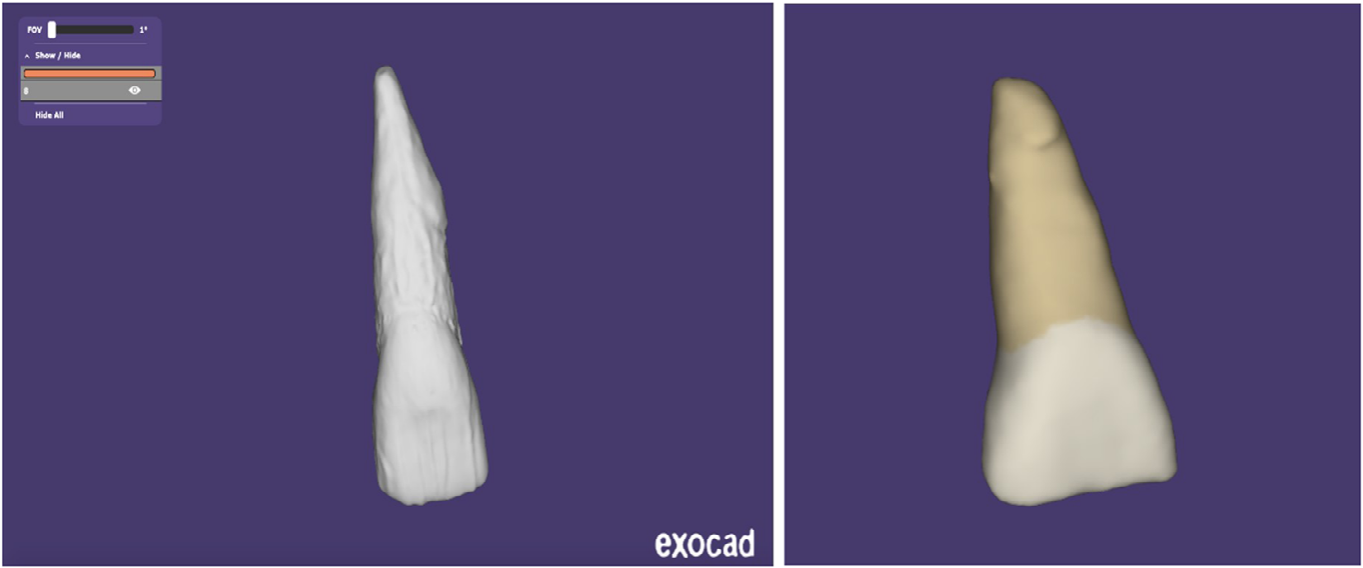
Exocad digital tooth library. The image on the left is an example of one of the final files being viewed in Exocad, an app students use to view the tooth ID files on smart phones, tablets, and computers. In 2021, the digital files were updated by painting them for a better anatomical representation (image shown on the right).

The digital tooth library was then distributed to dental and dental hygiene students via Google Drive to be visualized in a free 3D viewer app (Exocad Webview, Exocad, see Figure 2). This app is compatible with smart phones, tablets, and computers. Students accessed the digital library during their study sessions and faculty were able to demonstrate a specific tooth while everyone had the same view. In addition, the students had unlimited access to the digital files.

The course practical exam on tooth identification was 20 points of the course grade and students had to identify 20 teeth filling the blank in 20 min. The exam was in‐person and with one grader. A Qualtrics survey was distributed at the end of the semester to collect student's feedback about the use of the dental anatomy CAD file library.

## 3 RESULTS

The digital tooth morphology files supplemented the typical pre‐pandemic learning of dental anatomy. Despite the reduction in preclinical lab time compared to the 2019 fall semester, the dental students in the 2020 fall semester had a higher class average, a higher number of students receiving “A's,” and a lower number of students failing the course. Although the results are not statistically significant (*p* = 0.05), there was not a negative impact on the student‐learning outcomes (see Table 1).

**TABLE 1:** Fall 2019 and 2020 practical exam score comparison.

2019 practical exam (*n* = 120)	Score	2020 practical exam (*n* = 120)	Score
59	20	72	20
25	19	26	19
14	18	7	18
11	17	8	17
7	16	3	16
2	15	2	15
1	13	1	14
1	7	1	10
Mean (SD)	19.1 (2.1)	Mean (SD)	19.7 (1.8)

*Note*: *p*‐value from *t*‐test comparing two means (SD) = 0.05. Exam highest possible score = 20 points. Scores arranged in descending order.

Abbreviation: SD, standard deviation.

The Qualtrics survey data show that 91% of students stated the digital files are easy to use and they accessed the files after‐hours; 87% of students stated the review sessions helped them prepare for course exams; 95% of students indicated the digital files had a positive impact on final course grades; and 93% of students would like to see digital files available in future courses.

Since the 2020 fall semester, enhancements have been made to the digital tooth morphology library and continues to supplement student learning. In conclusion, the digital tooth morphology library is an effective study tool and can supplement in‐person learning without having a negative impact on student‐learning outcomes.
